# Supplementary material for: Secreted retropepsin-like enzymes are essential for stress tolerance and biofilm formation in Pseudomonas aeruginosa
Source: mBio. 2025 Jun 3;16(7):e00872-25. doi: 10.1128/mbio.00872-25 (PMC12239564; doi:10.1128/mbio.00872-25)
Supplement: Supplemental figures and tables — Figures S1-S9; Tables S1-S5. [file mbio.00872-25-s0001.pdf]

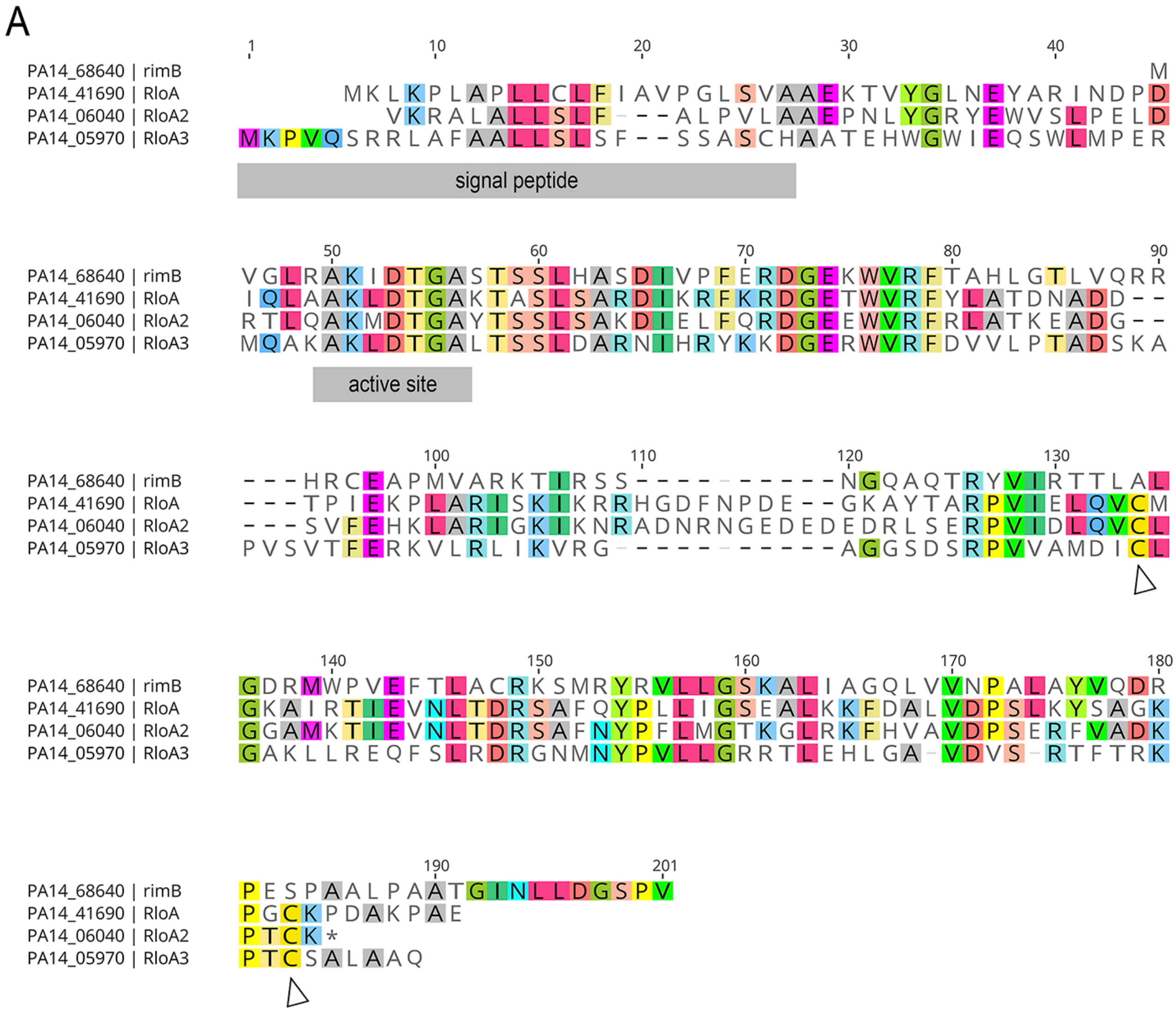

B

	PA14_68640 | rimB	PA14_41690 | RloA	PA14_06040 | RloA2	PA14_05970 | RloA3
PA14_68640 | rimB				
PA14_41690 | RloA	30.99%			
PA14_06040 | RloA2	31.88%	50.87%		
PA14_05970 | RloA3	30.37%	31.32%	32.95%	

Figure S1.- Sequence conservation between RimB and RloA proteases. **A.** Clustal Omega alignment highlighting the conserved signal peptide and conserved aspartic protease active site. Arrowheads point towards conserved disulfide-bond forming cysteine residues in RloA proteins. **B.** Protein sequence identity between pairwise combinations of RimB and RloA proteases.

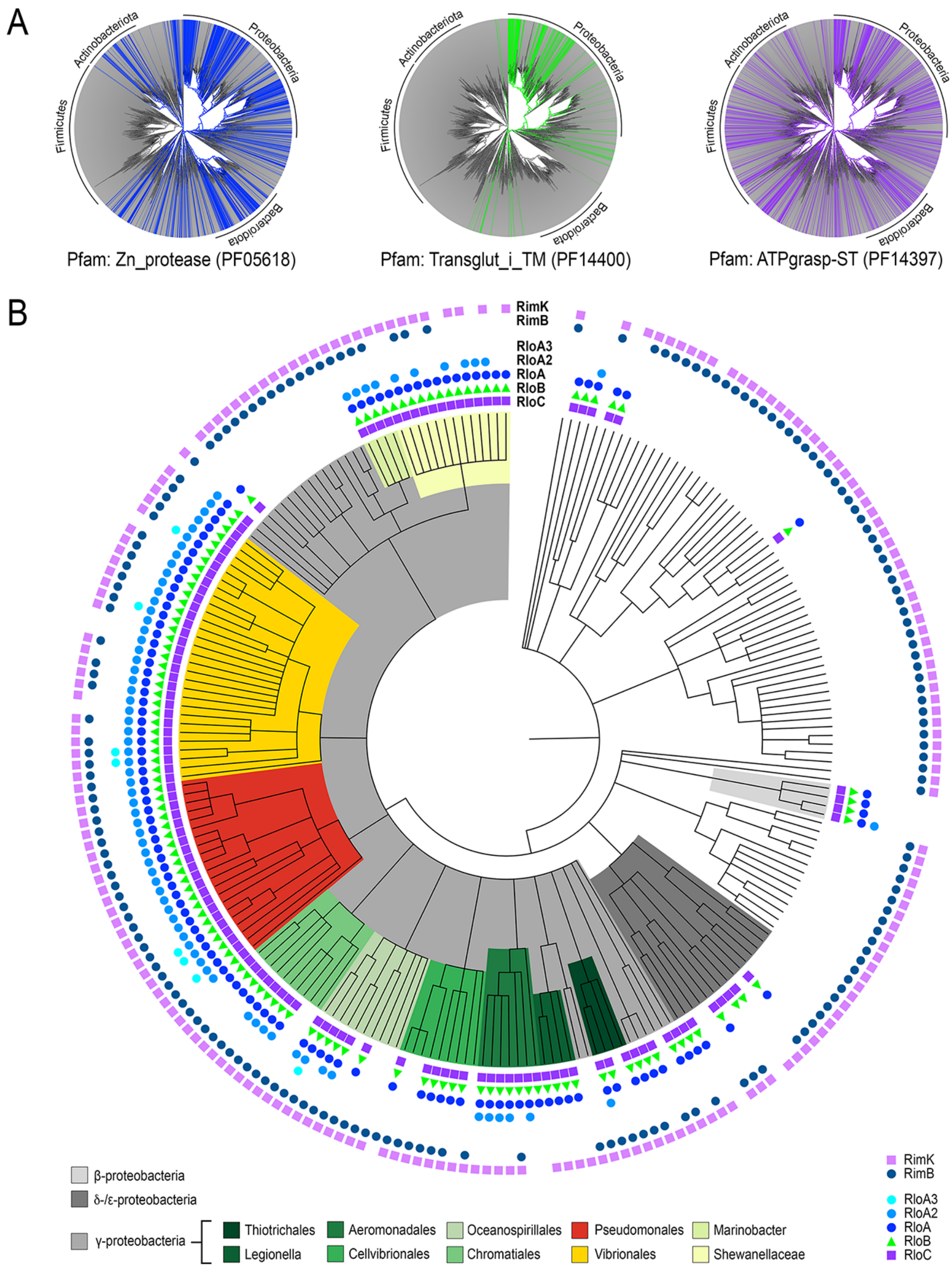

Figure S2.- Phylogenetic analysis of RloA proteases and the *rlo* operon. **A.** Phylogenetic conservation of Pfam groups for Zn_protease (PF05618, blue), Inactive transglutaminase fused to 7 transmembrane helices (PF1440, green) and ATP-grasp_ST (PF14397, purple) proteins is shown through AnnoTree plots (1). **B.** Plot showing the taxonomic distribution of RimB-RimK (outer circle) and Rlo proteins (inner circle). Plotted data was obtained by first surveying the EggNOG database (version 4.5.1) (2) with the RloA sequence as input. This retrieved a representative list of putative zinc protease sequences from bacteria (COG4067), which were then sorted according to their gene neighborhood into three groups: those adjacent to a *rimK* gene, those adjacent to *rloB* and *rloC* genes, and those with adjacent genes unrelated to the *rlo* or *rim loci*. The resulting gene clusters were then plotted using iTol (3). This analysis revealed that several organisms encode more than one RloA homologs containing N-terminal signal peptides (RloA2 and RloA3 in light blue circles). The fact that some organisms have more than one RloA homolog could either indicate emerging redundancy for RloA activity or further specialization within this enzyme family.

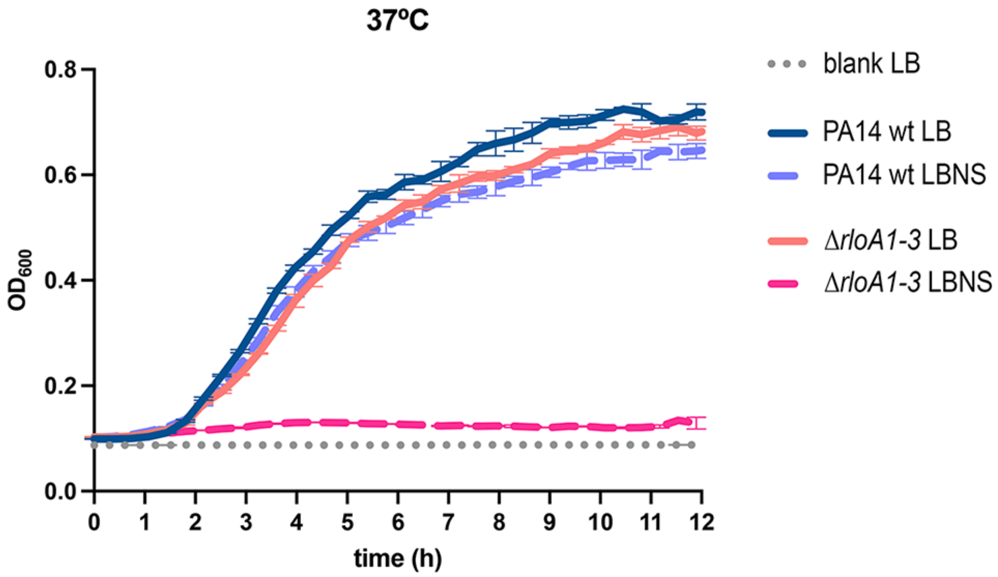

Figure S3.- Growth curve PA14 wild type and Δ*rloA1-3* mutants. Graph shows average OD^{600} measurements of three experimental replicas for the bacterial strains and experimental conditions indicated in the legend. Error bars show standard deviation. LB - Lysogeny Broth, LBNS - LB without salts.

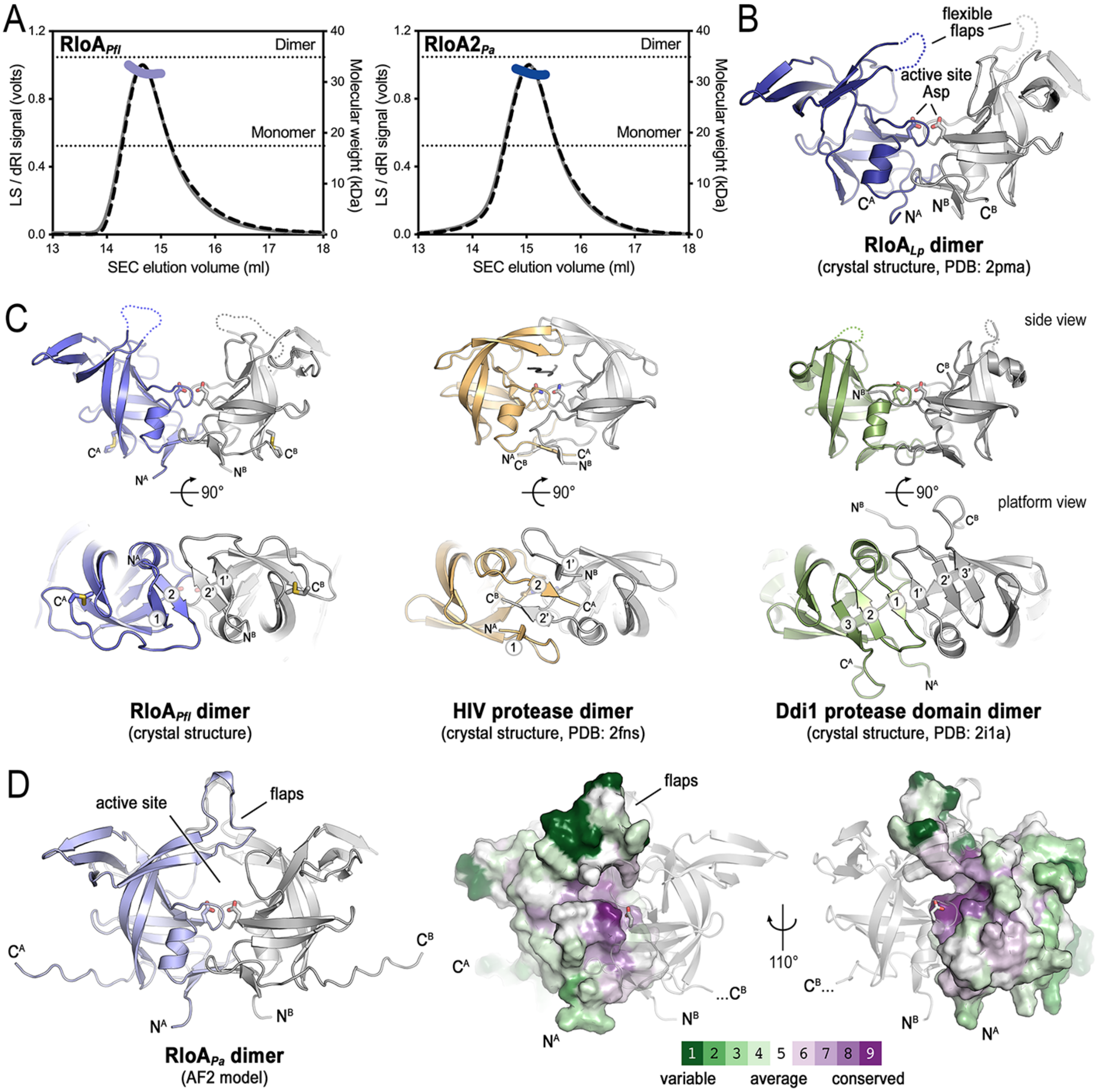

Figure S4.- Structural features of RloA proteases. **A.** SEC-MALS analysis of *P. fluorescens* RloA and *P. aeruginosa* RloA2. Normalized light scattering signal (grey line, right axis) and molar mass values (dashed line, left axis) were plotted against the SEC elution volume. Dotted lines show the theoretical molar mass ranges for monomeric and dimeric assemblies based on protein sequence. **B.** Crystal structure of a *Legionella pneumophila* RloA ortholog (PDB: 2pma). **C.** Crystal structures of *P. fluorescens* RloA (left), HIV protease (center, PDB: 2fns) and Ddi1 protease (right, PDB: 2i1a) dimers. Comparative side (top) and platform (bottom) views are shown. One of the protomers in each dimer has been colored in grey. **D.** ConSurf rendering of sequence conservation across a representative set of RloA proteases onto the AlphaFold2 model of *P. aeruginosa* RloA dimer. ConSurf scores are color coded as indicated. A ribbon view of the *P. aeruginosa* RloA AlphaFold2 model is shown to the left for reference.

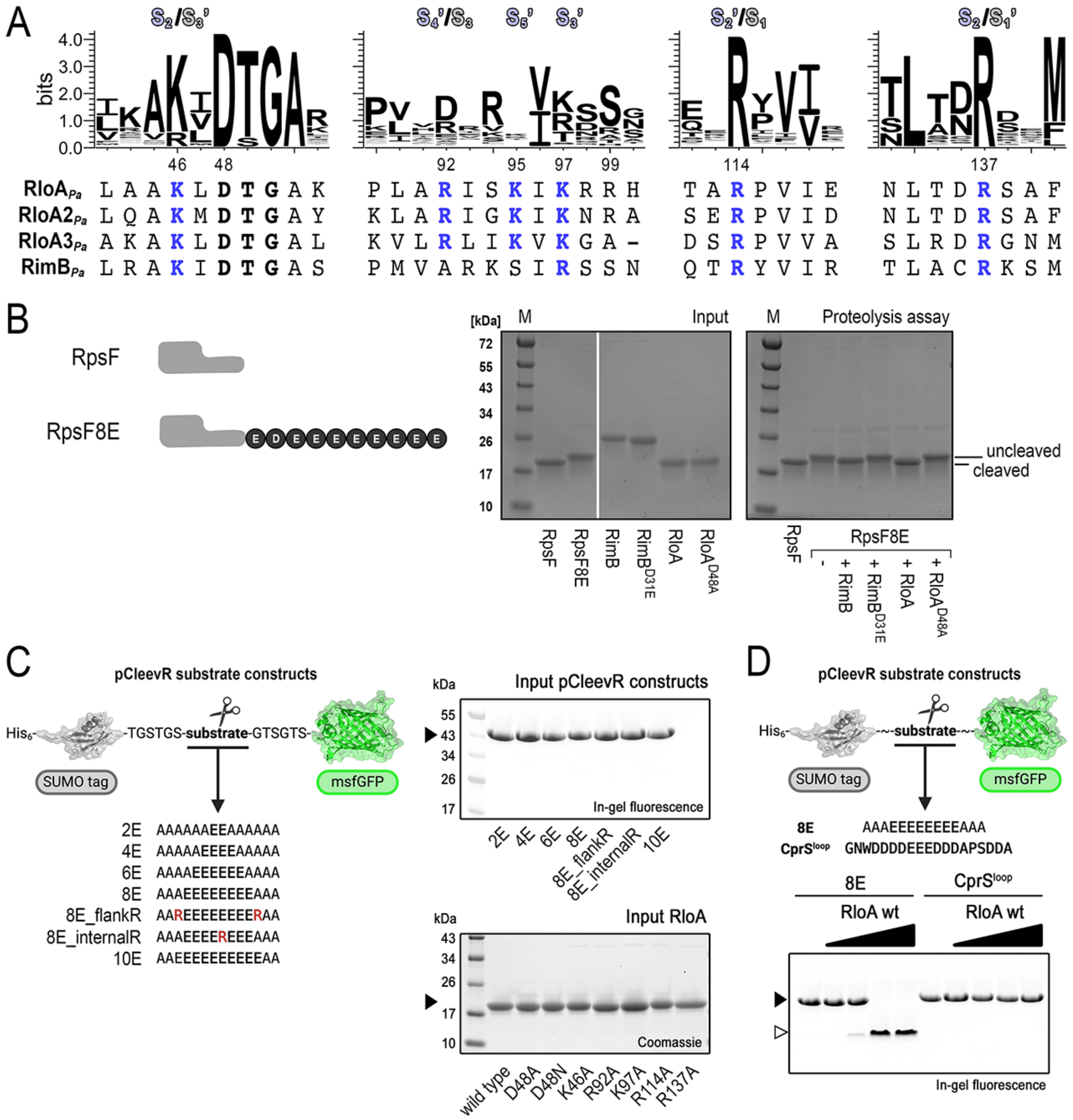

Figure S5.- A. Logos and alignments of RimB and RloA proteases highlighting the conserved active site (bold font) and residues involved in interactions with polyglutamate coordination in the AlphaFold2 model shown in Figure 3A (blue font). **B.** SDS-PAGE analysis showing the products of enzymatic reactions with the substrates schematically represented in the left panel and RimB or RloA proteins in their wild type or catalytically inactive mutant variants as indicated. Lines mark the positions of the cleaved and uncleaved substrates. **C.** Scheme illustrating the pCleevR polyglutamate substrates and input SDS-PAGE gels for experiments in Figure 3C-D. **D.** A sequence from CprS encoding a stretch of DE amino acids and their flanking residues was cloned into the pCleevR plasmid (top). Results from the incubation of RloA with the pCleevR-CprS^{loop} reporter protein are shown at the bottom. The pCleevR-8E construct was used as control. Empty and filled arrowheads mark the positions of cleaved and uncleaved substrates, respectively.

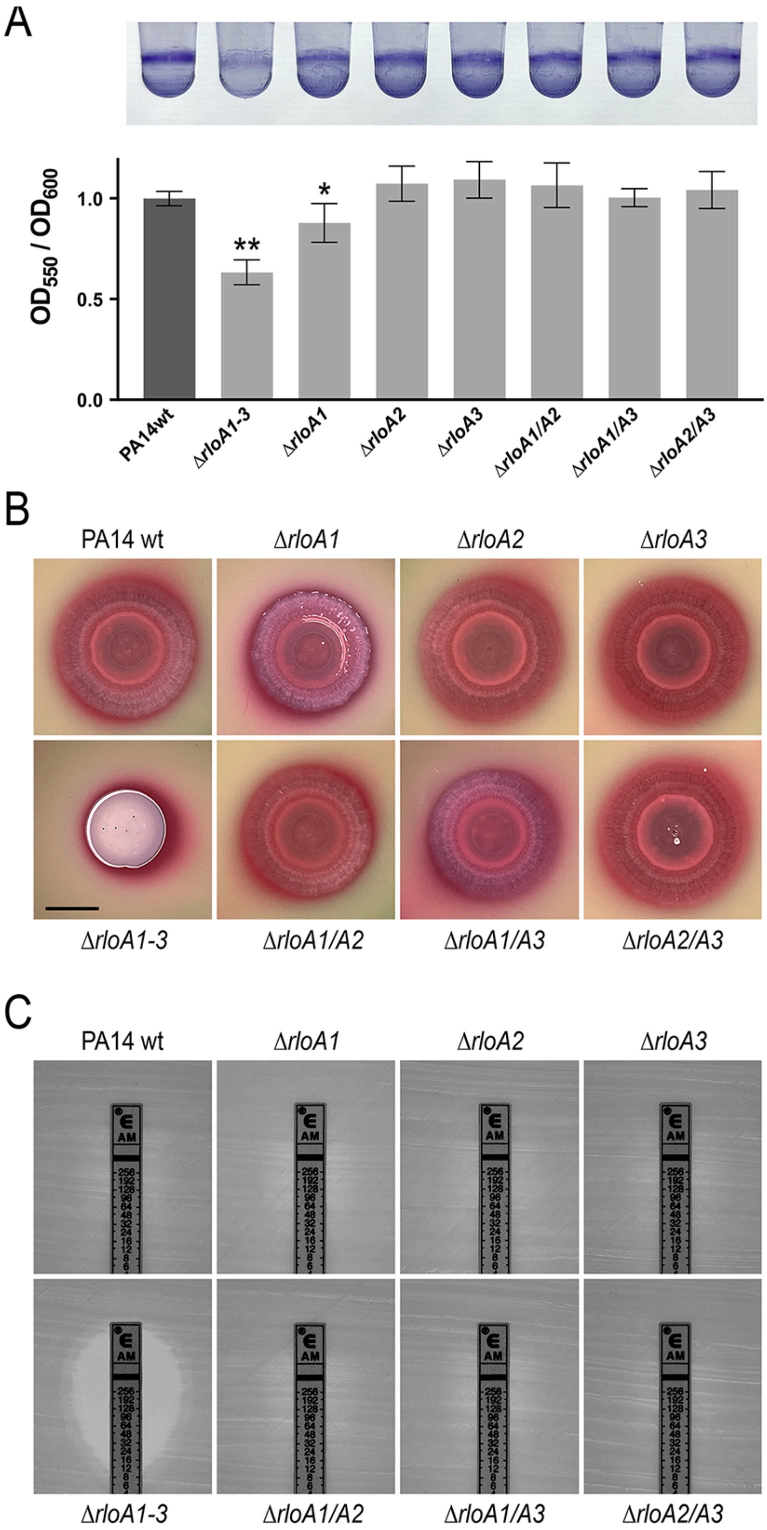

Figure S6.- Phenotypic characterization of *rloA* mutant combinations. A. Biofilm formation in PA14 wild type and combinations of RloA protease mutants was assessed through crystal violet staining of overnight cultures grown in 0.4% Arginine M63 media on PVC plates (top). Graph shows the normalized average intensity of crystal violet staining (OD^{550}) relative to the growth of the corresponding bacterial culture (OD^{600}), n=6. Error bars show standard deviation. Asterisks mark p values from paired two tailed t-tests against the PA14 wild type sample. *, p value <0.5. **, p value <0.0001. **B.** PA14 wild type and combinations of RloA protease mutant colonies grown in Congo Red 0.2% Glucose M63 agar plates. Scale bar represents 5 mm. **G.** Ampicillin ETEST strips tested on lawns of PA14 wild type and RloA protease mutant strains grown on Mueller-Hinton agar plates.

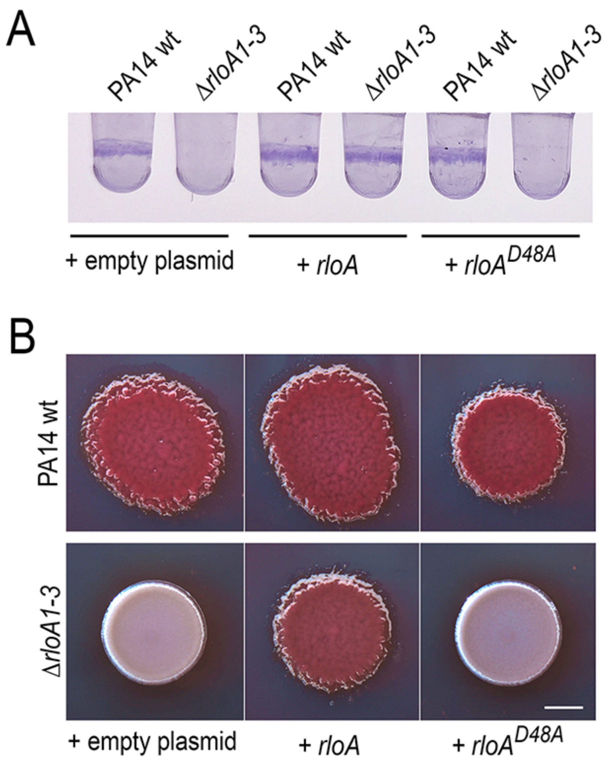

Figure S7.- Biofilm growth in Δ*rloA1-3* mutants upon expression of plasmid-encoded wild type and catalytically inactive *rloA*. A. Biofilm formation was assessed through crystal violet staining of overnight cultures grown on PVC plates in 0.2% arabinose M63 media with 25 µg/ml Gentamicin. **B.** Colonies of the indicated phenotypes were grown in Congo Red 0.4% Arginine M63 agar plates with 25 µg/ml Gentamicin. Scale bar represents 2 mm.

CELL WALL SYNTHESIS		
AMPICILLIN	PA14 WT	RESISTANT (>256.0)
AMPICILLIN	PA14 Δ*rloA1-3*	RESISTANT (16.0)
AUGMENTIN (AMOXICILLIN / CLAVULANIC ACID*)	PA14 WT	RESISTANT (128.0)
AUGMENTIN (AMOXICILLIN / CLAVULANIC ACID*)	PA14 Δ*rloA1-3*	RESISTANT (8.0)
PIPERACILLIN / TAZOBACT*	PA14 WT	SUSCEPTIBLE (<=8.0)
PIPERACILLIN / TAZOBACT*	PA14 Δ*rloA1-3*	SUSCEPTIBLE (<=8.0)
TICARCILLIN	PA14 WT	SUSCEPTIBLE (32.0)
TICARCILLIN	PA14 Δ*rloA1-3*	SUSCEPTIBLE (32.0)
CEFAZOLIN	PA14 WT	RESISTANT (>32.0)
CEFAZOLIN	PA14 Δ*rloA1-3*	RESISTANT (>32.0)
CEPHALEXIN	PA14 WT	RESISTANT (>256.0)
CEPHALEXIN	PA14 Δ*rloA1-3*	RESISTANT (32.0)
CEFTAZIDIME	PA14 WT	SUSCEPTIBLE (<=4.0)
CEFTAZIDIME	PA14 Δ*rloA1-3*	SUSCEPTIBLE (<=4.0)
BACITRACIN	PA14 WT	RESISTANT (>4.0)
BACITRACIN	PA14 Δ*rloA1-3*	RESISTANT (>4.0)
PROTEIN SYNTHESIS - 50S RIBOSOMAL SUBUNIT		
ERYTHROMYCIN	PA14 WT	RESISTANT (>4.0)
ERYTHROMYCIN	PA14 Δ*rloA1-3*	RESISTANT (>4.0)
CHLORAMPHENICOL	PA14 WT	RESISTANT (>16.0)
CHLORAMPHENICOL	PA14 Δ*rloA1-3*	INTERMEDIATE (16.0)
PROTEIN SYNTHESIS - 30S RIBOSOMAL SUBUNIT		
AMIKACIN	PA14 WT	SUSCEPTIBLE (<=16.0)
AMIKACIN	PA14 Δ*rloA1-3*	SUSCEPTIBLE (<=16.0)
GENTAMICIN	PA14 WT	SUSCEPTIBLE (<=2.0)
GENTAMICIN	PA14 Δ*rloA1-3*	SUSCEPTIBLE (<=2.0)
NEOMYCIN	PA14 WT	SUSCEPTIBLE (<=4.0)
NEOMYCIN	PA14 Δ*rloA1-3*	SUSCEPTIBLE (<=4.0)
TOBRAMYCIN	PA14 WT	SUSCEPTIBLE (<=4.0)
TOBRAMYCIN	PA14 Δ*rloA1-3*	SUSCEPTIBLE (<=4.0)
TETRACYCLINE	PA14 WT	RESISTANT (8.0)
TETRACYCLINE	PA14 Δ*rloA1-3*	RESISTANT (4.0)
DOXYCYCLINE	PA14 WT	RESISTANT (>2.0)
DOXYCYCLINE	PA14 Δ*rloA1-3*	RESISTANT (>2.0)
OXYTETRACYCLINE	PA14 WT	RESISTANT (4.0)
OXYTETRACYCLINE	PA14 Δ*rloA1-3*	RESISTANT (4.0)
NUCLEIC ACID SYNTHESIS		
OFLOXACIN	PA14 WT	SUSCEPTIBLE (0.25)
OFLOXACIN	PA14 Δ*rloA1-3*	SUSCEPTIBLE (0.5)
ENROFLOXACIN	PA14 WT	SUSCEPTIBLE (0.5)
ENROFLOXACIN	PA14 Δ*rloA1-3*	SUSCEPTIBLE (0.5)
CIPROFLOXACIN	PA14 WT	SUSCEPTIBLE (<=1.0)
CIPROFLOXACIN	PA14 Δ*rloA1-3*	SUSCEPTIBLE (<=1.0)
TRIMETHOPRIM / SULFAMETHOXAZOLE	PA14 WT	RESISTANT (4.0)
TRIMETHOPRIM / SULFAMETHOXAZOLE	PA14 Δ*rloA1-3*	RESISTANT (4.0)

Figure S8.- Antibiotic susceptibility panel. Table shows susceptibility of PA14 wild type and Δ*rloA1-3* mutants to a panel of antibiotics with different modes of action. β-lactamase inhibitors (asterisks) were included in some antibiotic test conditions.

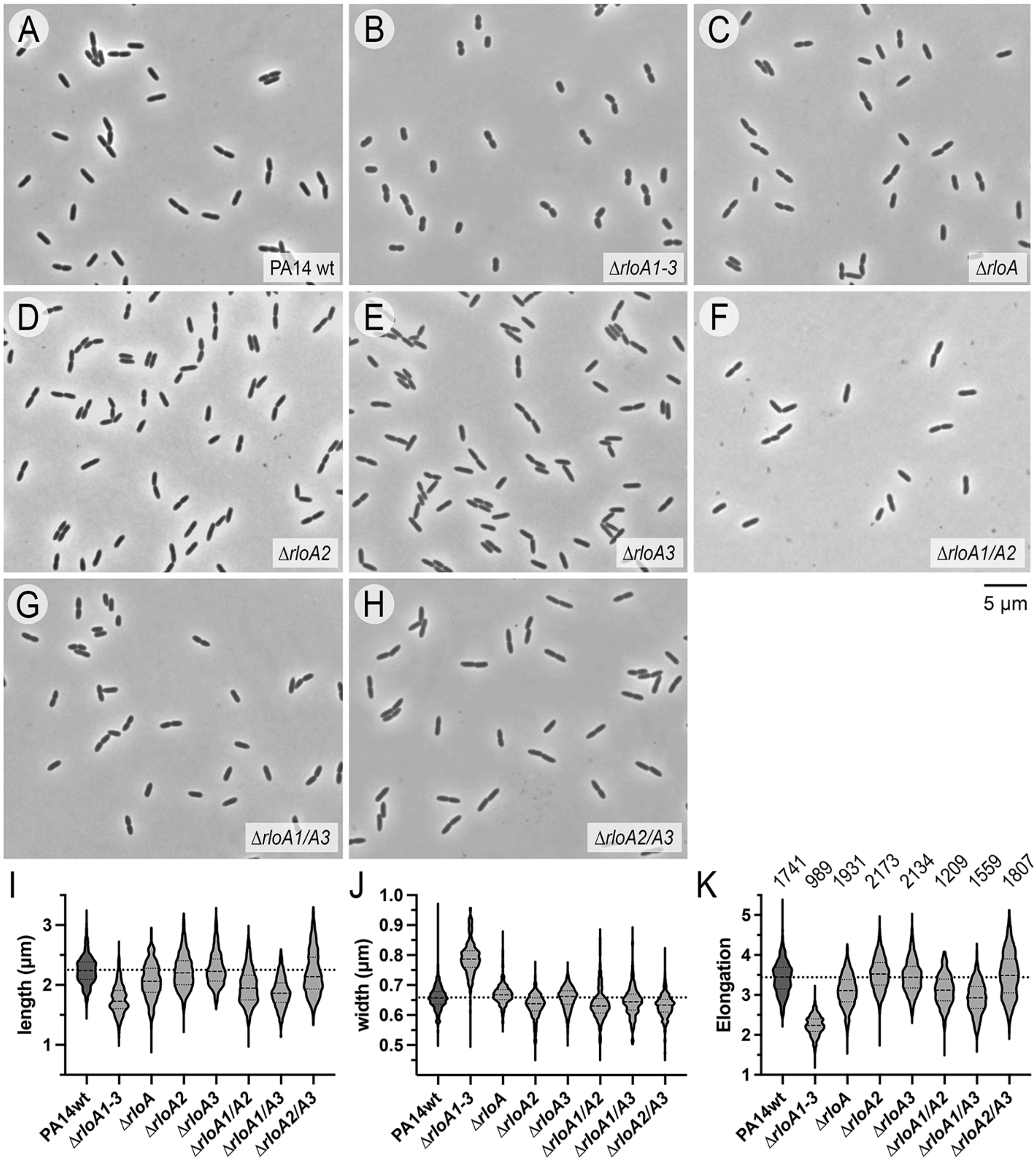

Figure S9.- Cell morphology in RloA protease mutants. A-H. Phase contrast images of PA14 wild type and RloA protease mutants of the indicated genotypes. Scale bar represents 5 µm. **I-K.** Violin plots showing quantification of cell length, width, and elongation. The number of samples analyzed for each strain is indicated on top of the graph in K. Quantification of measurements showed that individual Δ*rloA1* mutants, as well as double mutant combinations involving *rloA1* (Δ*rloA1/A2* and Δ*rloA1/A3* mutants) had a slight decrease in length. These results indicate that RloA1 might have a stronger dose-dependent requirement in comparison to the other RloA proteases.

Table S1. Crystalographic data collection and refinement statistics.*

Data Collection	rloA_Pfl	rloA2_Pa
Wavelength (Å)	0.9792	0.9792
Resolution range (Å)	45.0 - 2.28 (2.36 - 2.28)	61.6 - 2.40 (2.47 - 2.40)
Space group	$P\ 4_1$	$P\ 3_2\ 2\ 1$
Unit cell dimensions *a*, *b*, *c* (Å)	90.0 90.0 37.2	71.1 71.1 146.9
Unit cell angles α, β, γ (°)	90 90 90	90 90 120
Total reflections	148624 (13015)	207392 (15389)
Unique reflections	26253 (2402)	32573 (2681)
Multiplicity	5.7 (5.4)	6.4 (5.7)
Completeness (%)	98.91 (91.98)	99.84 (98.48)
Mean I / σ(I)	8.91 (1.42)	16.45 (1.06)
R_{merge}	0.09684 (1.069)	0.05016 (1.204)
CC1/2	0.994 (0.837)	0.999 (0.498)
**Refinement**		
Reflections used in refinement	13726 (1262)	17512 (1424)
Reflections used for R-free	1363 (124)	1750 (144)
R_{work} / R_{free}	21.69 / 24.69	22.02 / 24.82
Number of non-hydrogen atoms	1978	2153
macromolecules	1951	2136
solvent	27	17
RMS bonds (Å)	0.003	0.002
RMS angles (°)	0.63	0.53
Ramachandran favored (%)	96.81	96.56
Ramachandran allowed (%)	3.19	3.44
Ramachandran outliers (%)	0	0
Average B-factor	71.48	77.65
macromolecules	71.69	77.72
solvent	56.1	68.19

*Values in parentheses are for highest-resolution shell.

Table S2. Bacterial strains used in this study.

Strain	Genotype	Source/Reference	Additional information
E.coli T7 Express		New England Biolabs	
E. coli NEB® 5α		New England Biolabs	
E. coli S17.1 λpir$^+$		(4)	
P. aeruginosa UCBPP-PA14	wild type (PA14wt)	(5)	TaxID 208963
P. aeruginosa UCBPP-PA14	Δ*rloA*	This study	Generated with pEX18-*rloA* on PA14wt strain
P. aeruginosa UCBPP-PA14	Δ*rloA2*	This study	Generated with pEX18-*rloA2* on PA14wt strain
P. aeruginosa UCBPP-PA14	Δ*rloA3*	This study	Generated with pEX18-*rloA3* on PA14wt strain
P. aeruginosa UCBPP-PA14	Δ*rloA1/A2*	This study	Generated with pEX18-*rloA2* on Δ*rloA* strain
P. aeruginosa UCBPP-PA14	Δ*rloA1/A3*	This study	Generated with pEX18-*rloA3* on Δ*rloA* strain
P. aeruginosa UCBPP-PA14	Δ*rloA2/A3*	This study	Generated with pEX18-*rloA3* on Δ*rloA2* strain
P. aeruginosa UCBPP-PA14	Δ*rloA1-3*	This study	Generated with pEX18-*rloA3* on Δ*rloA1/A2* strain
P. aeruginosa UCBPP-PA14	Δ*rloB*	This study	Generated with pEX18-*rloB* on PA14wt strain
P. aeruginosa UCBPP-PA14	Δ*rloC*	This study	Generated with pEX18-*rloC* on PA14wt strain
P. aeruginosa UCBPP-PA14	Δ*rloABC*	This study	Generated with pEX18-*rloABC* on PA14wt strain
P. aeruginosa UCBPP-PA14	Δ*rloA1-3;* Δ*rloB*	This study	Generated with pEX18-*rloAB* on Δ*rloA1/A2* strain
P. aeruginosa UCBPP-PA14	Δ*rloA1-3;* Δ*rloC*	This study	Generated with pEX18-*rloC* on Δ*rloA1-3* strain
P. aeruginosa UCBPP-PA14	Δ*rloA1-3;* Δ*rloBC*	This study	Generated with pEX18-*rloABC* on Δ*rloA1/A2* strain
P. aeruginosa UCBPP-PA14	Δ*rimB*	This study	Generated with pEX18-*rimB* on PA14wt strain
P. aeruginosa UCBPP-PA14	Δ*rloA1-3;* Δ*rimB*	This study	Generated with pEX18-*rimB* on Δ*rloA1-3* strain
P. aeruginosa UCBPP-PA14	Δ*rloA1/A2;* Δ*rimB*	This study	Generated with pEX18-*rimB* on Δ*rloA1/A2* strain
P. aeruginosa UCBPP-PA14	Δ*rloA1/A3;* Δ*rimB*	This study	Generated with pEX18-*rimB* on Δ*rloA1/A3* strain
P. aeruginosa UCBPP-PA14	Δ*rloA2/A3;* Δ*rimB*	This study	Generated with pEX18-*rimB* on Δ*rloA2/A3* strain

Table S3. Plasmids used in this study.

Plasmid	Description	Source
His_6-SUMO-pET28	Fusion proteins cloned into this pET28-derived vector via BamHI-NotI render the protein of interest linked to an N-terminal His6-tagged small ubiquitin-like modifier (SUMO) cleavable by recombinant Ulp-1 protease. Antibiotic selection in 50 µg/ml Kanamycin	This study
His_6-SUMO-pET28-PA1768(23-179)	$RloA_{Pa}$ (aa 23-179 from PA1768) was PCR amplified and cloned via BamHI-NotI into His6-SUMO-pET28	This study
His_6-SUMO-pET28-PA1768(23-179) - D48A	Site directed mutagenesis was performed on His_6-SUMO-pET28-PA1768(23-179)	This study
His_6-SUMO-pET28-PA1768(23-179) - D48N	Site directed mutagenesis was performed on His_6-SUMO-pET28-PA1768(23-179)	This study
His_6-SUMO-pET28-PA1768(23-179) - K46A	Site directed mutagenesis was performed on His_6-SUMO-pET28-PA1768(23-179)	This study
His_6-SUMO-pET28-PA1768(23-179) - R92A	Site directed mutagenesis was performed on His_6-SUMO-pET28-PA1768(23-179)	This study
His_6-SUMO-pET28-PA1768(23-179) - K97A	Site directed mutagenesis was performed on His_6-SUMO-pET28-PA1768(23-179)	This study
His_6-SUMO-pET28-PA1768(23-179) - R114A	Site directed mutagenesis was performed on His_6-SUMO-pET28-PA1768(23-179)	This study
His_6-SUMO-pET28-PA1768(23-179) - R137A	Site directed mutagenesis was performed on His_6-SUMO-pET28-PA1768(23-179)	This study
His_6-SUMO-pET28-PA0462(18-169)	$RloA2_{Pa}$ (aa 18-169 from PA0462)was PCR amplified and cloned via BamHI-NotI into His6-SUMO-pET28	This study
His_6-SUMO-pET28-Pfl01_1762(20-178)	$RloA_{Pf}$ (aa 20-178 from Pfl01_1762) was PCR amplified and cloned via BamHI-NotI into His6-SUMO-pET28	This study
pETM11-RimB		(6)
pETM11-$RimB^{D31E}$		(7)
pETM11-RpsF		(6)
pETM11-RpsF8E		(7)
pCleevR	Antibiotic selection in 50 µg/ml Kanamycin	(10)
pCleevR-2E	2E gene fragment was cloned via BamHI-KpnI into pCleevR	This study
pCleevR-4E	4E gene fragment was cloned via BamHI-KpnI into pCleevR	This study
pCleevR-6E	6E gene fragment was cloned via BamHI-KpnI into pCleevR	This study
pCleevR-8E	8E gene fragment was cloned via BamHI-KpnI into pCleevR	This study
pCleevR-10E	10E gene fragment was cloned via BamHI-KpnI into pCleevR	This study
pCleevR-8E_flankR	8E_flankR gene fragment was cloned via BamHI-KpnI into pCleevR	This study
pCleevR-8E_internalR	8E_internalR gene fragment was cloned via BamHI-KpnI into pCleevR	This study
pCleevR-$CprS^{loop}$	$CprS^{loop}$ gene fragment was cloned via BamHI-KpnI into pCleevR	This study

Table S3 (cont.) Plasmids used in this study.

Plasmid	Description	Source
pJN105	Antibiotic selection in 15 µg/ml Gentamicin (*E.coli*) or 25-50 µg/ml Gentamicin (*Pseudomonas aeruginosa* PA14)	(8)
pJHA	Antibiotic selection in 15 µg/ml Gentamicin (*E.coli*) or 25-50 µg/ml Gentamicin (*Pseudomonas aeruginosa* PA14)	(9)
pJHA-*rloA*	*rloA* (PA14_41690) was PCR amplified and cloned via NdeI-EcoRI into pJHA	This study
pJHA-*rloA*^{D48A}	Site directed mutagenesis of pJHA-*rloA*	This study
pJHA-*rloA*^{K46A}	Site directed mutagenesis of pJHA-*rloA*	This study
pJHA-*rloA*^{R92A}	Site directed mutagenesis of pJHA-*rloA*	This study
pJHA-*rloA*^{K97A}	Site directed mutagenesis of pJHA-*rloA*	This study
pJHA-*rloA*^{R114A}	Site directed mutagenesis of pJHA-*rloA*	This study
pJHA-*rloA*^{R137A}	Site directed mutagenesis of pJHA-*rloA*	This study
pJHA-*rloA2*	*rloA2* (aa 156-234 from PA14_06040)[1] was PCR amplified and cloned via NdeI-EcoRI into pJHA	This study
pJHA-*rloA3*	*rloA3* (PA14_05970) was PCR amplified and cloned via NdeI-EcoRI into pJHA	This study
pJStrep	Strep-NheI-XbaI gene fragment was cloned via NheI-XbaI into pJN105. Antibiotic selection in 15 µg/ml Gentamicin (*E.coli*) or 25-50 µg/ml Gentamicin (*Pseudomonas aeruginosa* PA14)	This study
pJGCStrepII	msfGFP_CstrepII gene fragment was cloned via EcoRI-XbaI into pJN105. The EcoRI site at the polylinker was destroyed during cloning. pJGCStrepII can be used to generate C terminal Strep-tagged constructs (via EcoRI-HindIII) or proteins tagged with msfGFP at the N terminus and with Strep-tagII at the C terminus (via HindIII). Antibiotic selection in 15 µg/ml Gentamicin (*E.coli*) or 25-50 µg/ml Gentamicin (*Pseudomonas aeruginosa* PA14)	This study
pJStrep-*rloA*	*r*loA_CstrepII gene fragment was cloned via EcoRI-XbaI into pJN105	This study
pJStrep-*rloA*^{D48A}	Site directed mutagenesis of pJStrep-*rloA*	This study
pEX18Gm	Antibiotic selection in 15 µg/ml Gentamicin (*E.coli*) or 50 µg/ml Gentamicin (*Pseudomonas aeruginosa* PA14)	(4)
pEX18-*rloA*	*rloA* deletion allele was amplified through overlap extension PCR, then cloned via EcoRI-BamHI into pEX18-Gm	This study
pEX18-*rloA2*	*rloA2* deletion allele was amplified through overlap extension PCR, then cloned via EcoRI-BamHI into pEX18-Gm	This study
pEX18-*rloA3*	*rloA3* deletion allele was amplified through overlap extension PCR, then cloned via EcoRI-BamHI into pEX18-Gm	This study
pEX18-*rloB*	*rloB* deletion allele was amplified through overlap extension PCR, then cloned via EcoRI-BamHI into pEX18-Gm	This study
pEX18-*rloC*	*rloC* deletion allele was amplified through overlap extension PCR, then cloned via EcoRI-BamHI into pEX18-Gm	This study
pEX18-*rloABC*	*rlo operon* deletion allele was amplified through overlap extension PCR, then cloned via EcoRI-BamHI into pEX18-Gm	This study
pEX18-*rloAB*	*rloAB* deletion allele was amplified through overlap extension PCR, then cloned via EcoRI-BamHI into pEX18-Gm	This study
pEX18-*rimB*	*rimB* deletion allele was amplified through overlap extension PCR, then cloned via EcoRI-BamHI into pEX18-Gm	This study

[1] Note that PA14_06040 is annotated in Pseudomonas.com as a longer transcript that overlaps with gene PA14_06030 and is predicted to encode a protein without a signal peptide. However, orthologous genes in other *Pseudomonas* species (i.e. *P. aeruginosa* PA7 and *P. fluorescens* SBW25 at Pseudomonas.com or *P. aeruginosa* PAO1 at KEGG) are annotated as shorter transcripts encoding a signal peptide. We have considered PA14 RloA2 to encode an equivalent short protein of 169aa with a signal peptide. Here we refer to RloA2 as containing amino acids 156 to 234 of the annotated version of PA14_06040 available at Pseudomonas.com at the time this manuscript was written.

Table S4. Primers used in this study.

Name	Sequence	Purpose
PA1768-RloA-23BamHI-F	GAATTCGGATCCGCCGAAAAGACGGTCTACGGC	Insert for His_6-SUMO-pET28-PA1768(23-179)
PA1768-RloA-179stopNotI-R	CTCGAGGCGGCCGCTTACTCAGCAGGTTTTGCATCAGG	Insert for His_6-SUMO-pET28-PA1768(23-179)
PA0462s-RloA2-18-SUMO-F	ACAGATTGGTGGATCCGCCGAACCCAACCTCTACG	Insert for His_6-SUMO-pET28-PA0462(18-169)
PA0462s-RloA2-169-SUMO-R	TGCTCGAGTGCGGCCGCTTACTTGCAGGTGGGCTTGTC	Insert for His_6-SUMO-pET28-PA0462(18-169)
Pfl1762-20BamHI-F	CATATGGGATCCGCGGCGGGGGAAAAGACCGTGTACG	Insert for His_6-SUMO-pET28-Pfl01_1762(20-178)
Pfl1762-178stopNotI-R	TCTAGAGCGGCCGCTTACTCGGCGGTATGAGCGGCGATGG	Insert for His_6-SUMO-pET28-Pfl01_1762(20-178)
PA14_41690-RloA-1NdeI-F	GGATCCCATATGAAACTCAAGCCCTTAGCTCC	Insert for pJHA-*rloA*
PA14_41690-RloA-EcoRI-R	CGTCAGAATTCCTCAGCAGGTTTTGCATCAGGTTTGC	Insert for pJHA-*rloA*
rloA_D48A_s	CGCCAAGCTCGCCACCGGCGCCA	*rloA* D48A mutagenesis
rloA_D48A_as	TGGCGCCGGTGGCGAGCTTGGCG	*rloA* D48A mutagenesis
rloA_D48N_s	CCGCCAAGCTCAACACCGGCGCC	*rloA* D48N mutagenesis
rloA_D48N_as	GGCGCCGGTGTTGAGCTTGGCGG	*rloA* D48N mutagenesis
RloA_K46A_s	GCCGGTGTCGAGCGCGGCGGCCAGCTGG	*rloA* K46A mutagenesis
RloA_K46A_as	CCAGCTGGCCGCCGCGCTCGACACCGGC	*rloA* K46A mutagenesis
RloA_R92A_s	TTGATCTTGCTGATCGCCGCCAGGGGCTTCTC	*rloA* R92A mutagenesis
RloA_R92A_as	GAGAAGCCCCTGGCGGCGATCAGCAAGATCAA	*rloA* R92A mutagenesis
RloA_K97A_s	CCGTGGCGGCGCGCGATCTTGCTGATCCGCG	*rloA* K97A mutagenesis
RloA_K97A_as	CGCGGATCAGCAAGATCGCGCGCCGCCACGG	*rloA* K97A mutagenesis
RloA_R114A_s	CTCGATCACCGGAGCGGCGGTATAGGCC	*rloA* R114A mutagenesis
RloA_R114A_as	GGCCTATACCGCCGCTCCGGTGATCGAG	*rloA* R114A mutagenesis
RloA_R137A_s	GGGTATTGGAATGCACTTGCGTCGGTCAGGTTCACTTC	*rloA* R137A mutagenesis
RloA_R137A_as	GAAGTGAACCTGACCGACGCAAGTGCATTCCAATACCC	*rloA* R137A mutagenesis
PA14_06040-RloA2-F	AAGGAGATATACATATGAAGCGTGCCCTTGCCTTG	Insert for pJHA-*rloA2*
PA14_06040-RloA2-EcoRI-R	CGTCAGAATTCCTTGCAGGTGGGCTTGTCGGCGACG	Insert for pJHA-*rloA2*
PA14_05970-RloA3-F	AAGGAGATATACATATGAAGCCTGTGCAATCCCGTCG	Insert for pJHA-*rloA3*
PA14_05970-RloA3-EcoRI-R	CGTCAGAATTCCTGAGCGGCGAGCGCGCTGCAGG	Insert for pJHA-*rloA3*
PA14_41690-RloA-UF	CCATGATTACGAATTCCGAGGTACGCGAGGTCGA	Insert for pEX18-*rloA*
PA14_41690-RloA-UR	GATGCAGGTTGAGAGAGCGCATGTCGTAAGTCCTGGTTGGTGTCGCGTTCGGC	Insert for pEX18-*rloA*
PA14_41690-RloA-DF	ACGACATGCGCTCTCTCAACCTGCATC	Insert for pEX18-*rloA*
PA14_41690-RloA-DR	CGACTCTAGAGGATCCGGCTGTCGCGGAAGATCG	Insert for pEX18-*rloA*
rloA-seqF	ATCGACCCCGATCGCCTCA	Confirm allele deletion
rloA-seqR	CTGACGAAGGTCTCGACGTC	Confirm allele deletion
PA14_06040-RloA2-UF	CGACTCTAGAGGATCCGGAAGGGCACCCAGATCAG	Insert for pEX18-*rloA2*
PA14_06040-RloA2-UR	TTACTTGCAGGTGGGCTTGTCCAAGGCAAGGGCACGCTTCAC	Insert for pEX18-*rloA2*
PA14_06040-RloA2-DF	GACAAGCCCACCTGCAAGTAA	Insert for pEX18-*rloA2*
PA14_06040-RloA2-DR	CCATGATTACGAATTCGATCCGCACGCGCTCCTC	Insert for pEX18-*rloA2*

Table S4 (cont.) Primers used in this study.

Name	Sequence	Purpose
rloA2-seqF	CTTGGTGATCTCCAGGTC	Confirm allele deletion
rloA2-seqR	CTGCTCGCGGCTGAATAC	Confirm allele deletion
PA14_05970-RloA3-UF	CGACTCTAGAGGATCCGTCGTAGAGTGCTGACACTG	Insert for pEX18-*rloA3*
PA14_05970-RloA3-UR	CTACTGAGCGGCGAGCGCGCTACGGGATTGCACAGGCTTCAT	Insert for pEX18-*rloA3*
PA14_05970-RloA3-DF	AGCGCGCTCGCCGCTCAGTAG	Insert for pEX18-*rloA3*
PA14_05970-RloA3-DR	CCATGATTACGAATTCCTGATCCTCGGCTGCCTG	Insert for pEX18-*rloA3*
rloA3-seqF	GCAGCGACAGGCAATGTA	Confirm allele deletion
rloA3-seqR	ATGACCATGATCCCGCTG	Confirm allele deletion
PA14_41710-RloB-UF	CCATGATTACGAATTCCCTTAGCTCCCCTGCTCTGC	Insert for pEX18-*rloB*
PA14_41710-RloB-UR	CGCGGTCAGGTCTTTCCAGGTCTTCCAGATCGACATGGTTCAGTCCTTGAGCAGGGCCTT ATGCAGGTTGAGAGAGCGCATGTCGTTTACTCAGCAGGTTTTGCATCAGG	Insert for pEX18-*rloB*
PA14_41710-RloB-DF	CCATGTCGATCTGGAAGACCTGGAAAGACCTGACCGCG	Insert for pEX18-*rloB*
PA14_41710-RloB-DR	CGACTCTAGAGGATCCTCGGCAGGCGCAGCATGG	Insert for pEX18-*rloB*
rloB-seqF	GCGTGATAGAATGCGGCC	Confirm allele deletion
rloB-seqR	GACAACTCGTAGCAACCGG	Confirm allele deletion
PA14_41730-RloC-UF	CCATGATTACGAATTCGCGCTCCTATCTCGAGCA	Insert for pEX18-*rloC*
PA14_41730-RloC-UR	GCCGGGCTTCTTCATACCGGAAGCACGGGTTCAGTCCTTGAGCAGGGCCTTGAAGCGG	Insert for pEX18-*rloC*
PA14_41730-RloC-DF	CGTGCTTCCGGTATGAAGAAGCCCGGC	Insert for pEX18-*rloC*
PA14_41730-RloC-DR	CGACTCTAGAGGATCCCCCAGGGTGTCTACGGCC	Insert for pEX18-*rloC*
rloC-seqF	CGGTTCTGATCGCCCTGG	Confirm allele deletion
rloC-seqR	CCGCGCTACGAGGAGTTG	Confirm allele deletion
PA14_41690to41730-rloABC-UF	CGACTCTAGAGGATCCGGAACAGGAGCTTTCGGCC	Insert for pEX18-*rloABC*
PA14_41690to41730-rloABC-UR	TCAGCCCTGGTGCTGCTGGACAGCTAAGGGCTTGAGTTTCAT	Insert for pEX18-*rloABC*
PA14_41690to41730-rloABC-DF	GTCCAGCAGCACCAGGGCTGA	Insert for pEX18-*rloABC*
PA14_41690to41730-rloABC-DR	CCATGATTACGAATTCCCCTACTACACCTGGGTGG	Insert for pEX18-*rloABC*
PA14_41690+41710-rloAB-UR	TCAGTCCTTGAGCAGGGCCTTAGCTAAGGGCTTGAGTTTCAT	Insert for pEX18-*rloAB*
PA14_41690+41710-rloAB-DF	AAGGCCCTGCTCAAGGACTGA	Insert for pEX18-*rloAB*
PA14_41690+41710-rloAB-DR	CCATGATTACGAATTCGGGCGTTGAGTTCGAGGATCA	Insert for pEX18-*rloAB*
rloAB-seqR	GCTTCGTCAAACTGACGAG	Confirm allele deletion
rloAB-seqF	GGTGCGTTGAAATCCGAG	Confirm allele deletion
PA14_68640-RimB-BamHI-UF	CAGGTCGACTCTAGAGGATCCCGCAACTGGTGGATCTGCC	Insert for pEX18-*rimB*
PA14_68640-RimB-UR	AAACCGGACTCCCGACCATGCCCAGTTC	Insert for pEX18-*rimB*
PA14_68640-RimB-DF	CATGGTCGGGAGTCCGGTTTGAGCCATTCG	Insert for pEX18-*rimB*
PA14_68640-RimB-EcoRI-DR	TATGACCATGATTACGAATTCTCCAGCAGCTTGATCACC	Insert for pEX18-*rimB*
rimB-seqF	CTGGCCGTGCTGTTCATG	Confirm allele deletion
rimB-seqR	CCTGGACCATGATGTTGTGC	Confirm allele deletion

Table S5. Gene synthesized DNA fragments used in this study.

Name	Sequence	Purpose
2E	**TGGAAGCACGGGATCC**GCAGCTGCTGCGGCAGCCGAAGAGGCTGCA<br>GCTGCTGCAGCG**GGTACCAGCGGAACTTCTAG**	Insert for pCleevR-2E
4E	**TGGAAGCACGGGATCC**GCAGCTGCTGCGGCAGAGGAAGAGGAAGCA<br>GCTGCTGCAGCG**GGTACCAGCGGAACTTCTAG**	Insert for pCleevR-4E
6E	**TGGAAGCACGGGATCC**GCAGCTGCTGCGGAAGAGGAAGAGGAAGAA<br>GCTGCTGCAGCG**GGTACCAGCGGAACTTCTAG**	Insert for pCleevR-6E
8E	**TGGAAGCACGGGATCC**GCAGCTGCTGAGGAAGAGGAAGAGGAAGAA<br>GAGGCTGCAGCG**GGTACCAGCGGAACTTCTAG**	Insert for pCleevR-8E
10E	**TGGAAGCACGGGATCC**GCAGCTGAAGAGGAAGAGGAAGAGGAAGA<br>AGAGGAGGCAGCG**GGTACCAGCGGAACTTCTAG**	Insert for pCleevR-10E
8E_flankR	**TGGAAGCACGGGATCC**GCAGCTAGAGAAGAAGAGGAAGAGGAAGA<br>GGAAAGAGCTGCA**GGTACCAGCGGAACTTCTAG**	Insert for pCleevR-8E_flankR
8E_internalR	**TGGAAGCACGGGATCC**GCAGCTGCTGAGGAAGAGGAAAGAGAAGAA<br>GAGGCTGCAGCG**GGTACCAGCGGAACTTCTAG**	Insert for pCleevR-8E_internalR
CprS^{loop}	**TGGAAGCACGGGATCC**GGTAACTGGGACGATGATGACGAAGAGGAA<br>GATGACGATGCGCCGTCTGACGATGCG**GGTACCAGCGGAACTTCTAG**	Insert for pCleevR-CprS^{loop}
Strep-NheI-XbaI	**GCTAGC**CATATGCCCGGGGAGCTCGAATTCAAGCTTGAGCTCCCT**TGG<br>AGCCACCCGCAGTTCGAGAAG**TAA**TCTAGA**	Insert for pJStrep
msfGFP_CstrepII	**TTGGGCTAGCGAATT**GAATTC**ATG**AGCAAAGGTGAAGAACTGTTTACC<br>GGCGTTGTGCCGATTCTGGTGGAACTGGATGGTGATGTGAATGGCCA<br>TAAATTTAGCGTTCGTGGCGAAGGCGAAGGTGATGCGACCAACGGTA<br>AACTGACCCTGAAATTTATTTGCACCACCGGTAAACTGCCGGTTCCGTG<br>GCCGACCCTGGTGACCACCCTGACCTATGGCGTTCAGTGCTTTAGCCG<br>CTATCCGGATCATATGAAACGCCATGATTTCTTTAAAAGCGCGATGCCG<br>GAAGGCTATGTGCAGGAACGTACCATTAGCTTCAAAGATGATGGCACC<br>TATAAAACCCGTGCGGAAGTTAAATTTGAAGGCGATACCCTGGTGAAC<br>CGCATTGAACTGAAAGGTATTGATTTTAAAGAAGATGGCAACATTCTG<br>GGTCATAAACTGGAATATAATTTCAACAGCCATAATGTGTATATTACCG<br>CCGATAAACAGAAAAATGGCATCAAAGCGAACTTTAAAATCCGTCACA<br>ACGTGGAAGATGGTAGCGTGCAGCTGGCGGATCATTATCAGCAGAAT<br>ACCCCGATTGGTGATGGCCCGGTGCTGCTGCCGGATAATCATTATCTG<br>AGCACCCAGAGCAAACTGAGCAAAGATCCGAATGAAAAACGTGATCA<br>TATGGTGCTGCTGGAATTTGTTACCGCCGCGGGCATTACCCACGGTAT<br>GGATGAACTGTATAAAGGCAGCAAGCTTTCTGCGTGGTCTCACCCGCA<br>GTTCGAAAAA**TAA**TCTAGA**GCGGCCGCCA**	Insert for pJGCStrepII
rloA_CstrepII	**TTGGGCTAGCGAATTCATG**AAACTCAAGCCCTTAGCTCCCCTGCTCTGC<br>CTATTCATCGCCGTACCAGGCCTGAGCGTAGCCGCCGAAAAGACGGT<br>CTACGGCCTGAACGAATACGCGCGCATCAACGACCCGGACATCCAGC<br>TGGCCGCCAAGCTCGACACCGGCGCCAAGACCGCCTCCCTCAGCGCC<br>CGCGACATCAAGCGTTTCAAGCGCGACGGCGAAACCTGGGTGCGCTT<br>CTACCTGGCCACCGACAACGCCGACGACACCCCGATCGAGAAGCCCC<br>TGGCGCGGATCAGCAAGATCAAGCGCCGCCACGGCGACTTCAACCCC<br>GACGAGGGCAAGGCCTATACCGCCCGTCCGGTGATCGAGCTGCAGG<br>TCTGCATGGGCAAGGCGATTCGCACCATCGAAGTGAACCTGACCGAC<br>CGAAGTGCATTCCAATACCCGCTTTTGATCGGCTCGGAGGCCTTGAAG<br>AAATTCGACGCACTGGTCGATCCGAGTTTGAAATACTCGGCCGGGAAA<br>CCCGGCTGCAAACCTGATGCAAAACCTGCTGAGTCTGCGTGGTCTCAC<br>CCGCAGTTCGAAAAA**TAATCTAGAGCGGCCGCCA**	Insert for pJStrep-*rloA*

REFERENCES

1. Mendler K, Chen H, Parks DH, Lobb B, Hug LA, Doxey AC. 2019. AnnoTree: visualization and exploration of a functionally annotated microbial tree of life. Nucleic Acids Res 47:4442–4448.

2. Huerta-Cepas J, Szklarczyk D, Forslund K, Cook H, Heller D, Walter MC, Rattei T, Mende DR, Sunagawa S, Kuhn M, Jensen LJ, von Mering C, Bork P. 2016. eggNOG 4.5: a hierarchical orthology framework with improved functional annotations for eukaryotic, prokaryotic and viral sequences. Nucleic Acids Res 44:D286–D293.

3. Letunic I, Bork P. 2024. Interactive Tree of Life (iTOL) v6: recent updates to the phylogenetic tree display and annotation tool. Nucleic Acids Res 52:W78-W82.

4. Hmelo LR, Borlee BR, Almblad H, Love ME, Randall TE, Tseng BS, Lin C, Irie Y, Storek KM, Yang JJ, Siehnel RJ, Howell PL, Singh PK, Tolker-Nielsen T, Parsek MR, Schweizer HP, Harrison JJ. 2015. Precision-engineering the *Pseudomonas aeruginosa* genome with two-step allelic exchange. Nat Protoc 10:1820–1841.

5. Rahme LG, Stevens EJ, Wolfort SF, Shao J, Tompkins RG, Ausubel FM. 1995. Common virulence factors for bacterial pathogenicity in plants and animals. Science 268:1899–1902.

6. Little RH, Grenga L, Saalbach G, Howat AM, Pfeilmeier S, Trampari E, Malone JG. 2016. Adaptive remodeling of the bacterial proteome by specific ribosomal modification regulates *Pseudomonas* Infection and niche colonisation. Plos Genet 12:e1005837.

7. Little RH, Chandra G, Saalbach G, Martins C, Thompson CMA, Malone JG. 2025. A retropepsin-like bacterial protease regulates ribosome modification and polypeptide production. J Biol Chem 108329. (Online ahead of print)

8. Newman JR, Fuqua C. 1999. Broad-host-range expression vectors that carry the l-arabinose-inducible *Escherichia coli araBAD* promoter and the *araC* regulator. Gene 227:197–203.

9. Lormand JD, Kim S-K, Walters-Marrah GA, Brownfield BA, Fromme JC, Winkler WC, Goodson JR, Lee VT, Sondermann H. 2021. Structural characterization of NrnC identifies unifying features of dinucleases. eLife 10:e70146.

10. Cooley RB, Smith TJ, Leung W, Tierney V, Borlee BR, O’Toole GA, Sondermann H. 2015. Cyclic di-GMP-regulated periplasmic proteolysis of a *Pseudomonas aeruginosa* type Vb secretion system substrate. J Bacteriol 198:66–76.
